# Comparison of microRNA Expression Profile in Chronic Myeloid Leukemia Patients Newly Diagnosed and Treated by Allogeneic Hematopoietic Stem Cell Transplantation

**DOI:** 10.3389/fonc.2020.01544

**Published:** 2020-09-04

**Authors:** Juliana Ravelli Baldassarre Martins, Leonardo Nazario de Moraes, Sarah Santiloni Cury, Juliane Dadalto, Juliana Capannacci, Robson Francisco Carvalho, Célia Regina Nogueira, Newton Key Hokama, Paula de Oliveira Montandon Hokama

**Affiliations:** ^1^Department of Internal Medicine, São Paulo State University (UNESP-FMB), Botucatu, Brazil; ^2^Department of Bioprocesses and Biotechnology, São Paulo State University (UNESP-FCA), Botucatu, Brazil; ^3^Department of Structural and Functional Biology, São Paulo State University (UNESP-IBB), Botucatu, Brazil

**Keywords:** miRNAs, chronic myeloid leukemia, allogeneic hematopoietic stem cell transplantation, biomarkers, Philadelphia chromosome

## Abstract

Chronic myeloid leukemia (CML) results from a translocation between chromosomes 9 and 22, which generates the Philadelphia chromosome. This forms BCR/ABL1, an active tyrosine kinase protein that promotes cell growth and replication. Despite great progress in CML treatment in the form of tyrosine kinase inhibitors, allogeneic-hematopoietic stem cell transplantation (allo-HSCT) is currently used as an important treatment alternative for patients resistant to these inhibitors. Studies have shown that unregulated expression of microRNAs, which act as oncogenes or tumor suppressors, is associated with human cancers. This contributes to tumor formation and development by stimulating proliferation, angiogenesis, and invasion. Research has demonstrated the potential of microRNAs as biomarkers for cancer diagnosis, prognosis, and therapeutic targets. In the present study, we compared the circulating microRNA expression profiles of 14 newly diagnosed patients with chronic phase-CML and 14 Philadelphia chromosome-negative patients after allo-HSCT. For each patient, we tested 758 microRNAs by reverse transcription quantitative polymerase chain reaction (RT-qPCR) analysis. The global expression profile of microRNAs revealed 16 upregulated and 30 downregulated microRNAs. Target genes were analyzed, and key pathways were extracted and compared. Bioinformatics tools were used to analyze data. Among the downregulated miRNA target genes, some genes related to cell proliferation pathways were identified. These results reveal the comprehensive microRNA profile of CML patients and the main pathways related to the target genes of these miRNAs in cytogenetic remission after allo-HSCT. These results provide new resources for exploring stem cell transplantation-based CML treatment strategies.

## Introduction

Chronic myeloid leukemia (CML) is a myeloproliferative disease accounting for ~20% of diagnosed adult cases (1, 2). CML was the first human malignant disease to be linked to a chromosomal abnormality. A translocation between chromosomes 9q34 and 22q11 generates the Philadelphia chromosome. This encodes the BCR/ABL1 oncoprotein, an active tyrosine kinase protein that is the main driver of CML pathogenesis (2, 3). There have been great developments and improvements in anticancer targeted therapy associated with CML (4). Imatinib was the first member of a class of small molecules that prevent tyrosine kinase activity to be developed, and acts by binding to the BCR-ABL1 protein. Tyrosine kinase inhibitors (TKIs) act upon the interaction between the BCR-ABL1 oncoprotein and ATP, blocking cellular proliferation of the malignant clone. However, ~2% of patients become resistant to TKIs. Allogeneic-hematopoietic stem cell transplantation (allo-HSCT) is the only curative treatment for CML and provides an important alternative for TKI-resistant or advanced phase CML patients (2). However, the mortality and morbidity of this method, as well as a lack of suitable donors, limits the application of allo-HSCT (5).

The natural course of CML begins in the chronic phase and progresses to the blast phase, passing through the accelerated phase. The transformation mechanisms involved with this process are varied and not yet fully understood. The interruption of differentiation, genomic instability, shortening of telomeres, and loss of tumor suppressor functions are among the steps of this transformation that have already been described (6, 7).

Recent advancements in gene expression profiling technology have demonstrated that microRNAs (miRNAs) are promising prognostic predictors of different types of cancers. miRNAs, which modulate post-transcriptional gene expression, are 18–25-nucleotide non-coding RNAs (8–10). They regulate gene expression and modify cancer processes such as differentiation, proliferation, and apoptosis. Previous studies have suggested that miRNAs play important roles in regulating angiogenesis and metastasis (11, 12). Additionally, miRNAs are very stable molecules in the blood, suggesting that they can be applied as molecular markers (13).

Bioinformatics is an important research approach that can be applied for understanding gene regulation pathways. Cancer bioinformatics is an emerging field that integrates knowledge from cancer and information technology. Integrating cancer research and bioinformatics is important for advancing the diagnosis, prognosis, and treatment of cancer (14). Additionally, bioinformatics analyses have contributed to the identification of candidate genes and miRNA-mRNA target pairs (15).

This study was conducted to determine the profiles of miRNAs and their target genes in CML patients treated with allo-HSCT. These profiles were then compared to those of a newly diagnosed and untreated patient group.

## Patients and Methods

The study was previously approved by the Research Ethics Committee of the Dr. Amaral Carvalho Hospital, Jahu, SP, Brazil (CEPHAC-−2.917.389). The patients provided informed consent to participate in the study, in accordance with the Declaration of Helsinki.

### Patients and Samples

A total of 28 patients diagnosed with chronic phase-CML and treated at the Dr. Amaral Carvalho Hospital were included in the study. The patients were separated into two groups: (1) 14 patients newly diagnosed with Philadelphia chromosome-positive (Ph+) CML who had not been treated with TKIs; and (2) 14 patients who achieved complete cytogenetic remission (Philadelphia chromosome-negative) post-allo-HSCT. Patient characteristics are described in Table 1. The present study used a leukocyte pool of 14 healthy blood donors as a control group. To determine the miRNA profiles of each patient group, the control group was compared to the patient groups to determine which miRNAs were upregulated or downregulated. All transplanted patients received BuCy-2 as a conditioning regimen and cyclosporine and methotrexate as graft-vs.-host disease prophylaxis (16, 17).

**Table 1:** Patient clinical data.

**Patient**		**Gender**		**Age**		**BCR-ABL (%)**			**Breakpoint**
**Untreated group**									
1		M		61		92			b2a2
2		M		52		100			b2a2
3		M		35		100			b2a2
4		F		54		100			b2a2
5		M		46		73			b3a2
6		M		72		100			b2a2
7		F		80		100			b3a2
8		M		67		100			b3a2
9		M		59		40			b2a2
10		M		60		82			b2+b3
11		F		52		100			b3a2
12		M		37		100			b3a2
13		F		53		100			b3a2
14		M		72		100			b3a2
**Patients**	**Gender**	**Age**	**HSCT indication**	**Source**	**DAT**	**BCR-ABL (%)**	**Breakpoint**	**Mutation**	**Follow up**
**Hematopoietic stem cell transplantation group**									
1	M	37	Disease progression	BM	224	1.60	b2a2	Absence	Relapsed
2	F	44	Failed therapy	BM	231	1,20	b3a2	Absence	Relapsed
3	F	03	Disease progression	BM	208	0.20	b3a2	_	Relapsed
4	F	63	FC	BM	1569	0.20	b3a2	Absence	Relapsed
5	M	37	Failed therapy	BM	110	0.50	b2a2	_	Relapsed
6	M	43	FC	BM	2450	0.30	b3a2	_	MMR
7	M	48	Disease progression	PBSC	80	0.75	b3a2	Absence	MMR
8	M	35	Failed therapy	BM	2352	0.03	b3a2	Absence	Relapsed
9	M	42	Failed therapy	PBSC	3561	0.01	b2a2	_	Relapsed
10	F	42	Disease progression	PBSC	3708	0.01	b3a2	_	MMR
11	F	56	FC	BM	3932	0.03	b2a2	Absence	MMR
12	M	32	FC	PBSC	3242	0.08	b2a2	_	Relapsed
13	M	21	FC	BM	1949	0.30	b3a2	Absence	Relapsed
14	M	58	Disease progression	BM	41	0.02	b2a2	_	MMR

*DAT, days after transplantation; BM, bone marrow; PBSC, peripheral blood stem cell; (-), BCR-ABL mutation test was not performed; MMR, Major molecular response (BCR-ABL1 transcript level ≤ 0.1%)*.

### Total RNA Isolation and RT-qPCR

Total RNA isolation was performed using a QIAamp® RNA Blood Mini Kit (QIAGEN, Hilden, Germany) with 5 mL peripheral blood. RNA integrity and quantity were evaluated by NanoDrop (Thermo Scientific, Waltham, MA, USA) and RNA 2100 Bioanalyzer (Agilent Technologies, Santa Clara, CA, USA) according to the manufacturer's instructions.

The total RNA was reverse-transcribed using a Taqman MicroRNA Reverse Transcription Kit, and a TaqMan^TM^ MGB probe was used for real-time qPCR according to the manufacturer's instructions. RT-qPCR and TaqMan® Low Density Array Human MicroRNA Arrays A v2.0 and B v3.0 (ABIV®, Life Technologies, Carlsbad, CA, USA) were performed on a ViiA7 platform (ABIV®) following the manufacturer's instructions. miRNAs were quantified using the comparative Ct-method (18). Each Human Pool Set contains 377 unique human miRNAs, three internal controls, and one negative control. A total of 758 miRNAs were analyzed.

### Bioinformatic Analysis

**Figure 1:**
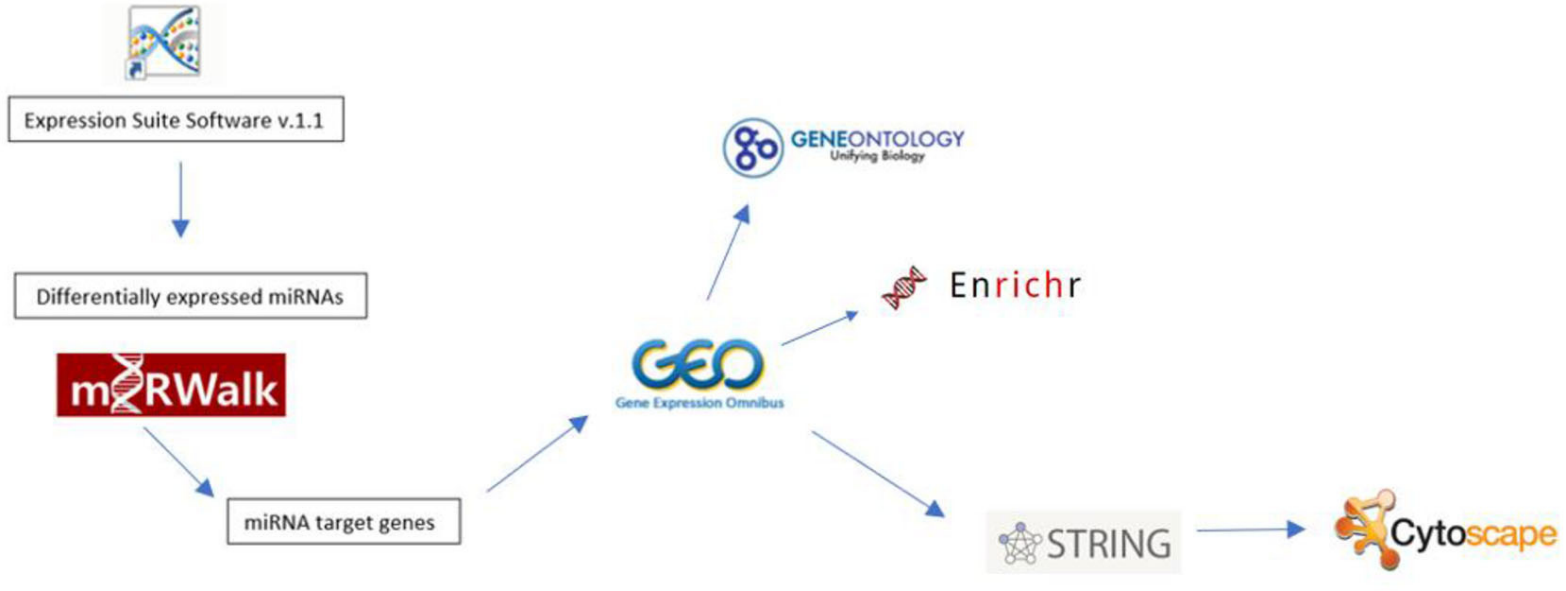
Flowchart showing the experimental procedure.

The study followed the experimental procedure detailed in Figure 1. Expression Suite Software Version 1.1 Program was used to identify differentially expressed miRNAs. To identify possible differentially expressed miRNA targets, we conducted RT-qPCR using miRWalk 2.0, which includes target prediction data generated by different algorithms (including own algorithm) (19). The following algorithms were selected: miRWalk, miRDB, Micro T4, miRanda, RNAhybrid, and Targetscan. Only targets predicted by at least three of the selected algorithms were accepted. We then verified whether the predicted targets have been identified as being differentially expressed in CML using microarray data available from Gene Expression Omnibus (https://www.ncbi.nlm.nih.gov/geo), accession number GSE43225. Microarray data were analyzed using the GEO2R script(https://www.ncbi.nlm.nih.gov/geo/geo2r/).

We considered genes to be differentially expressed when they showed a fold-change of at least 1.5. Gene Ontology (GO) (http://www.geneontology.org/) was used to search for enriched terms among differentially expressed genes, accepting only terms with *P* ≤ 0.05 and using Bonferroni's correction. Differentially expressed genes related to upregulated and downregulated miRNAs were analyzed according to EnrichR (https://amp.pharm.mssm.edu/Enrichr/) for enrichment analysis. Reactome was used for analysis. To assess the protein-protein interaction (PPI) network based on a list of genes, the online tool STRING Version 11.0 (https://string-db.org/) was utilized. Experiments, databases, co-expression data, neighborhood, and co-occurrence were considered active interaction sources. The minimum required interaction score was 0.700. Finally, we used Cytoscape Version 3.8.0 (http://www.cytoscape.org) software to visualize the enrichment results. Network nodes represent genes, while edges represent protein-protein associations.

### Statistical Analysis

Comparative C_T_ analysis was used to quantify miRNA gene expression. The differences were estimated by Student's *t*-test. Values of *P* < 0.05 were considered statistically significant.

## Results

### Differentially Expressed miRNAs

The miRNA expression data set is available in the NCBI GEO database (accession number GSE148567). A total of 758 miRNAs were analyzed by RT-qPCR in peripheral blood samples from 14 newly diagnosed patients and untreated chronic phase CML patients, and 14 patients in cytogenetic remission after allo-HSCT. According to the cut-off criteria (fold-change ≤ 0.5 and fold-change ≥ 2.0), 46 differentially expressed miRNAs were identified. Sixteen (34.8%) miRNAs were upregulated, and thirty (65.2%) were downregulated (Table 2). Among them, miR-1260a was the most downregulated miRNA, whereas miR-409-3p was the most upregulated miRNA. The main functions of all differentially expressed miRNAs are listed in Table 3.

**Table 2:** Dysregulated miRNAs in chronic myeloid leukemia.

**miR**	**miRBase ID**	**FC**	***P*-value**
hsa-miR-1260a	MIMAT0005911	0.013	0.000
hsa-miR-27a-3p	MIMAT0000084	0.082	0.000
hsa-miR-140-3p	MIMAT0004597	0.083	0.000
mmu-miR-374b-5p	MIMAT0003727	0.105	0.000
hsa-miR-143-3p	MIMAT0000435	0.150	0.000
hsa-miR-181c-5p	MIMAT0000258	0.159	0.000
hsa-miR-26b-5p	MIMAT0000083	0.160	0.000
hsa-miR-212-3p	MIMAT0000269	0.164	0.000
hsa-miR-29c-3p	MIMAT0000681	0.172	0.000
hsa-miR-26a-1-3p	MIMAT0004499	0.180	0.000
hsa-miR-181a-5p	MIMAT0000256	0.189	0.000
hsa-miR-19a-3p	MIMAT0000073	0.224	0.008
hsa-miR-363-3p	MIMAT0000707	0.243	0.001
hsa-miR-30d-5p	MIMAT0000245	0.252	0.000
hsa-miR-10a-5p	MIMAT0000253	0.259	0.014
hsa-miR-29a-3p	MIMAT0000086	0.291	0.000
hsa-miR-16-5p	MIMAT0000069	0.291	0.002
hsa-miR-486-5p	MIMAT0002177	0.292	0.029
hsa-miR-345-5p	MIMAT0000772	0.310	0.000
hsa-miR-26a-5p	MIMAT0000082	0.318	0.000
hsa-miR-18a-3p	MIMAT0002891	0.329	0.000
hsa-miR-27b-3p	MIMAT0000419	0.331	0.003
hsa-miR-374a-5p	MIMAT0000727	0.343	0.009
hsa-miR-362-5p	MIMAT0000705	0.350	0.000
hsa-let-7g-5p	MIMAT0000414	0.402	0.000
hsa-miR-324-3p	MIMAT0000762	0.417	0.000
hsa-miR-550a-5p	MIMAT0004800	0.417	0.003
hsa-miR-125a-3p	MIMAT0004602	0.444	0.046
hsa-miR-106b-5p	MIMAT0000680	0.477	0.001
hsa-miR-191-5p	MIMAT0000440	0.496	0.000
hsa-miR-15b-3p	MIMAT0004586	2.005	0.039
hsa-miR-328-3p	MIMAT0000752	2.179	0.013
hsa-miR-222-3p	MIMAT0000279	2.233	0.000
hsa-miR-139-5p	MIMAT0000250	2.338	0.039
hsa-miR-92a-3p	MIMAT0000092	2.492	0.002
hsa-miR-628-3p	MIMAT0003297	2.589	0.010
hsa-miR-150-5p	MIMAT0000451	2.748	0.034
hsa-miR-574-3p	MIMAT0003239	2.764	0.001
hsa-miR-484	MIMAT0002174	2.820	0.000
hsa-miR-127-3p	MIMAT0000446	3.969	0.007
hsa-miR-146a-5p	MIMAT0000449	3.973	0.000
hsa-miR-193a-5p	MIMAT0004614	4.513	0.000
hsa-miR-342-3p	MIMAT0000753	5.070	0.000
hsa-miR-7-1-3p	MIMAT0004553	5.650	0.000
mmu-miR-134-5p	MIMAT0000146	6.473	0.002
hsa-miR-409-3p	MIMAT0001639	10.905	0.004

*FC, fold-change; miR, microRNA*.

**Table 3:** Functions of dysregulated miRNAs in chronic myeloid leukemia.

**miR Name**	**Dysregulation in cancer**	**References**
hsa-miR-1260a	Down-regulated in follicular B-cell lymphoma	(20)
hsa-miR-27a-3p	Involved in tumor growth: cell proliferation and cell invasion	(21)
hsa-miR-140-3p	Chemoresistance in osteosarcoma and colon cancer	(22)
mmu-miR-374b-5p	Inhibits cell migration, proliferation and invasion in cervical cancer	(23)
hsa-miR-143-3p	Low expression level contributes to tumor development, differentiation, proliferation, invasion and metastasis	(24)
hsa-miR-181c-5p	Inhibits chemoresistance in chronic myelocytic leukemia	(25)
hsa-miR-26b-5p	Down-regulated in breast cancer	(26)
hsa-miR-212-3p	Inhibits cell proliferation and promotes apoptosis	(27)
hsa-miR-29c-3p	Deregulated in hematological malignances	(28)
hsa-miR-26a-1-3p	Tumor suppressor	(29)
hsa-miR-181a-5p	Downregulation in resistance to imatinib	(30, 31)
hsa-miR-19a-3p	Potential biomarker for CML	(32)
hsa-miR-363-3p	Tumor suppressor in gastric cancer	(33)
hsa-miR-30d-5p	Downregulation in resistance to imatinib	(30, 31)
hsa-miR-10a-5p	Biomarker of drug response in CML	(34)
hsa-miR-29a-3p	Deregulated in hematological malignances	(28)
hsa-miR-16-5p	Regulation of cell cycle and apoptosis in myeloid leukemogenesis	(35)
hsa-miR-486-5p	Expression increased in erythroid differentiation in CML	(36)
hsa-miR-345-5p	Tumor suppressor in pancreatic cancer	(37)
hsa-miR-26a-5p	Tumor suppressor	(29)
hsa-miR-18a-3p	Potential biomarker for CML	(32)
hsa-miR-27b-3p	Oncogene; expression increased in lymphoma	(38)
hsa-miR-374a-5p	Promotes proliferation and migration of transformed mesenchymal stem cells	(39)
hsa-miR-362-5p	Induces apoptosis resistance and cell proliferation in gastric cancer	(40)
hsa-let-7g-5p	Downregulated in Burkitt's lymphoma	(41)
hsa-miR-324-3p	Overexpression promotes cell growth and decreases apoptosis	(42)
hsa-miR-550a-5p	Tumor suppressor	(43)
hsa-miR-125a-3p	Induces apoptosis in pancreatic cancer	(44)
hsa-miR-106b-5p	Inhibits metastasis and invasion colorectal cancer cells	(45)
hsa-miR-191-5p	Disregulated in human gliobastoma tissues	(46)
hsa-miR-15b-3p	High expression in poor prognosis for hepatocellular carcinoma	(47)
hsa-miR-328-3p	CML progression	(48)
hsa-miR-222-3p	Cancer development as oncomiR or as oncosuppressor	(49)
hsa-miR-139-5p	Antimetastic and anti-oncogenic activity	(50)
hsa-miR-92a-3p	Higher levels in acute myeloid leukemia and acute lymphoblastic leukemia	(51)
hsa-miR-628-3p	Inhibits proliferation of acute myeloid leukemia cells	(52, 53)
hsa-miR-150-5p	CML progression; CML biomarker	(54)
hsa-miR-574-3p	Tumor suppressor in ovarian cancer	(55)
hsa-miR-484	Highly expressed in breast cancer patients	(56)
hsa-miR-127-3p	Tumor suppressors in gastric cancer	(57)
hsa-miR-146a-5p	Development and maintenance of neoplastic processes	(58)
hsa-miR-193a-5p	Low expression in lung cancer	(59)
hsa-miR-342-3p	Suppresses acute myeloid leukemia cell proliferation	(60)
hsa-miR-7-1-3p	Up-regulated in metastatic prostate cancer	(61)
mmu-miR-134-5p	Cancer cell proliferation	(62)
hsa-miR-409-3p	Tumor suppressor in endometrial carcinoma cells	(63)

*Fc, fold change; miR, microRNA*.

### MiRNA Target Genes

Upregulated and downregulated miRNAs were analyzed in miRWalk to identify the miRNA target genes. Using microarray analysis, 1,069 genes were identified.

### Gene Expression

We evaluated whether the identified target genes were previously differentially expressed in CML using available microarray data in the Gene Expression Omnibus. The microarray data were analyzed using GEO2R script. The identified genes were compared to our results. Of the 822 genes related to downregulated miRNA, 789 (96%) were also identified among the Gene Expression Omnibus microarray data genes. Among the genes associated with upregulated miRNAs, 247 genes were found in the patients included in the experiment, and 234 (95%) were found in the data searched.

### Pathway Enrichment Analysis and PPI Network Construction

**Figure 2:**
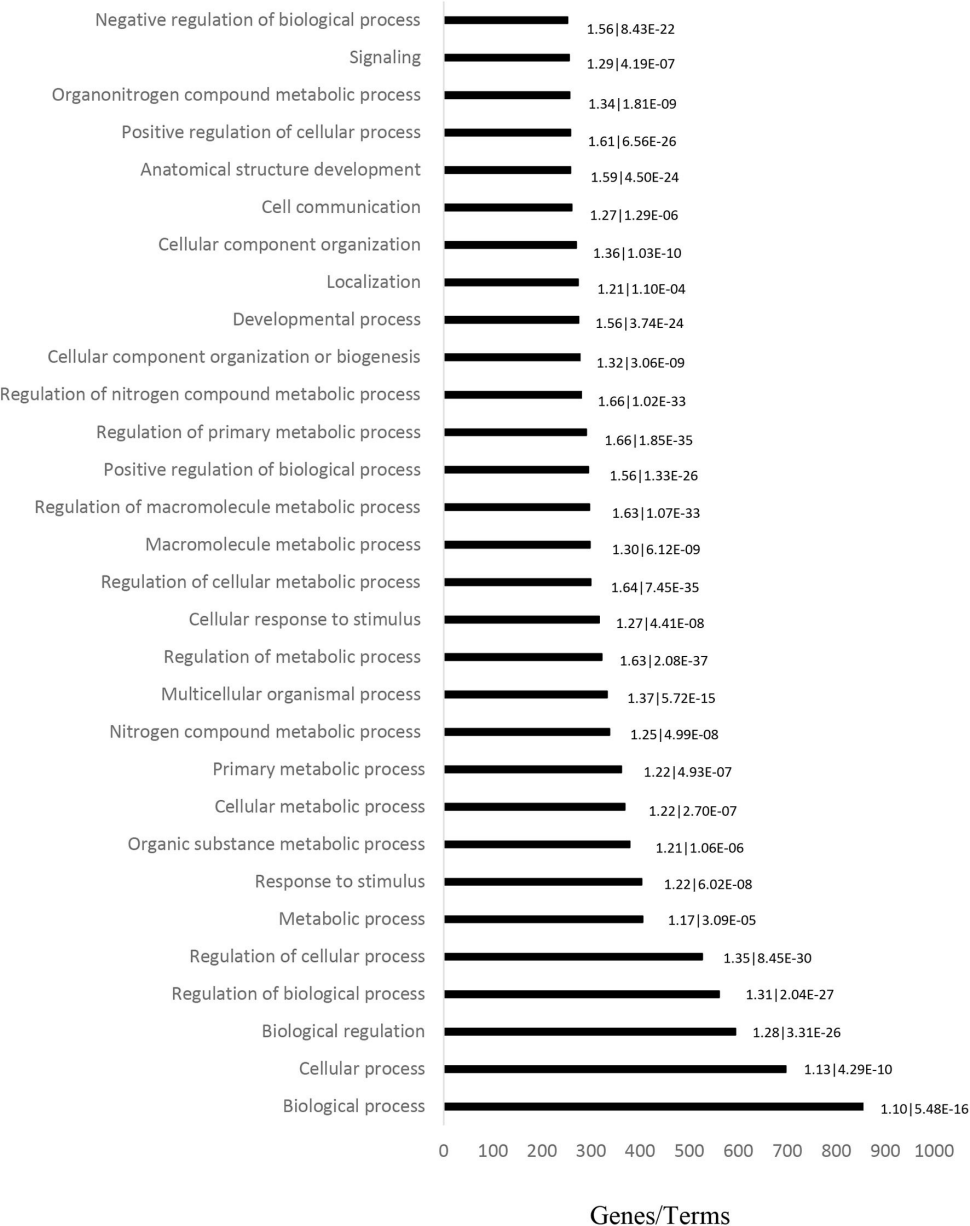
Ontology analysis of differentially expressed genes in CML patient blood samples, performed to determine the main altered pathways. Horizontal bars represent the number of genes found in each term. The fold-enrichment and *P*-values are displayed on the sides of the horizontal bars, and are separated by a vertical line.

GO analysis (including Molecular Function, Biological Process and Cellular Component) was performed on 1,069 target genes. A total of 461 results were obtained from GO analysis. The top 30 terms are shown in Figure 2.

**Figure 3:**
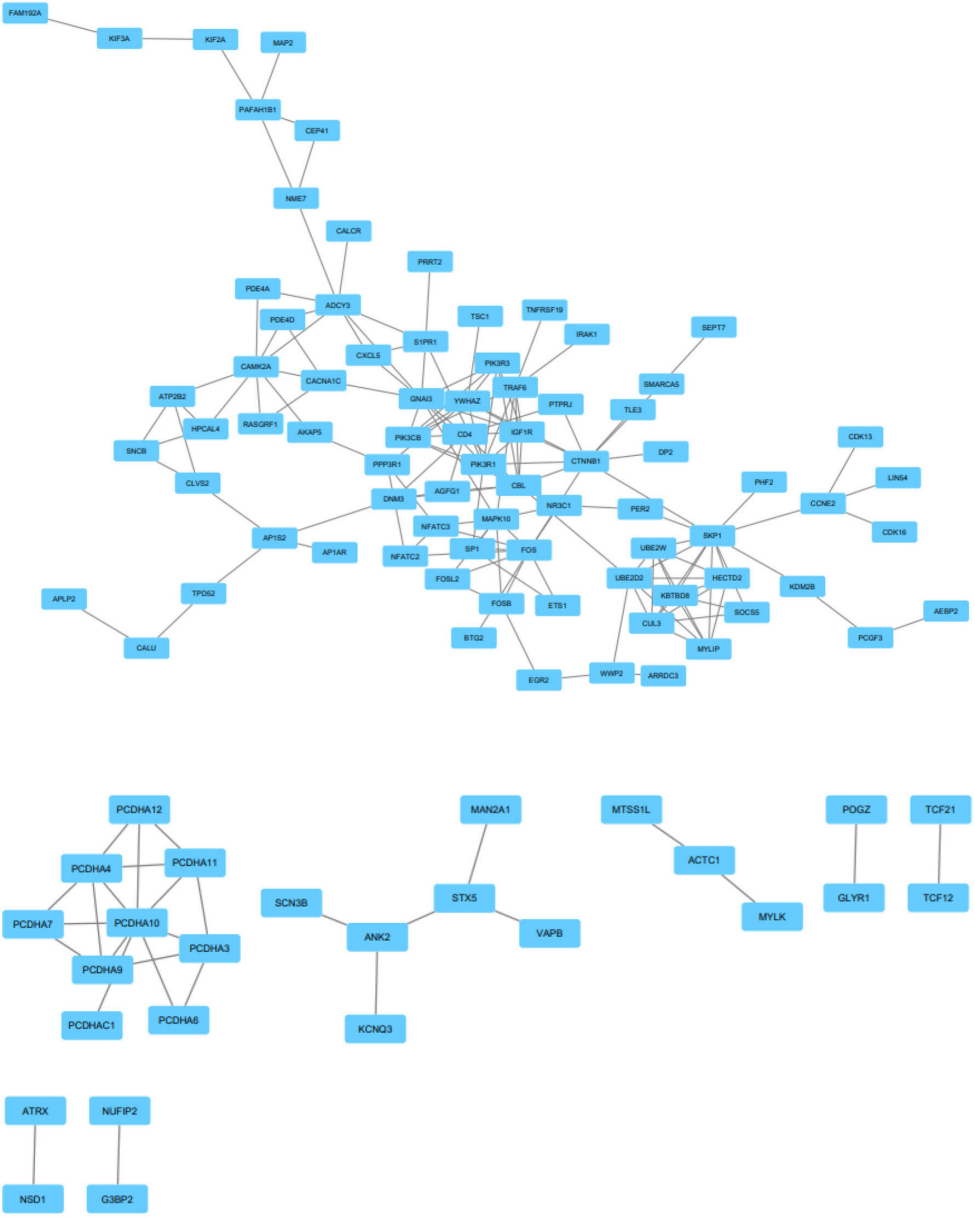
Protein-protein interaction network analysis of the target genes of upregulated miRNAs. Gene interactions were constructed using the STRING online database and Cytoscape. Network nodes represent genes, and the edges represent protein-protein associations.

**Figure 4:**
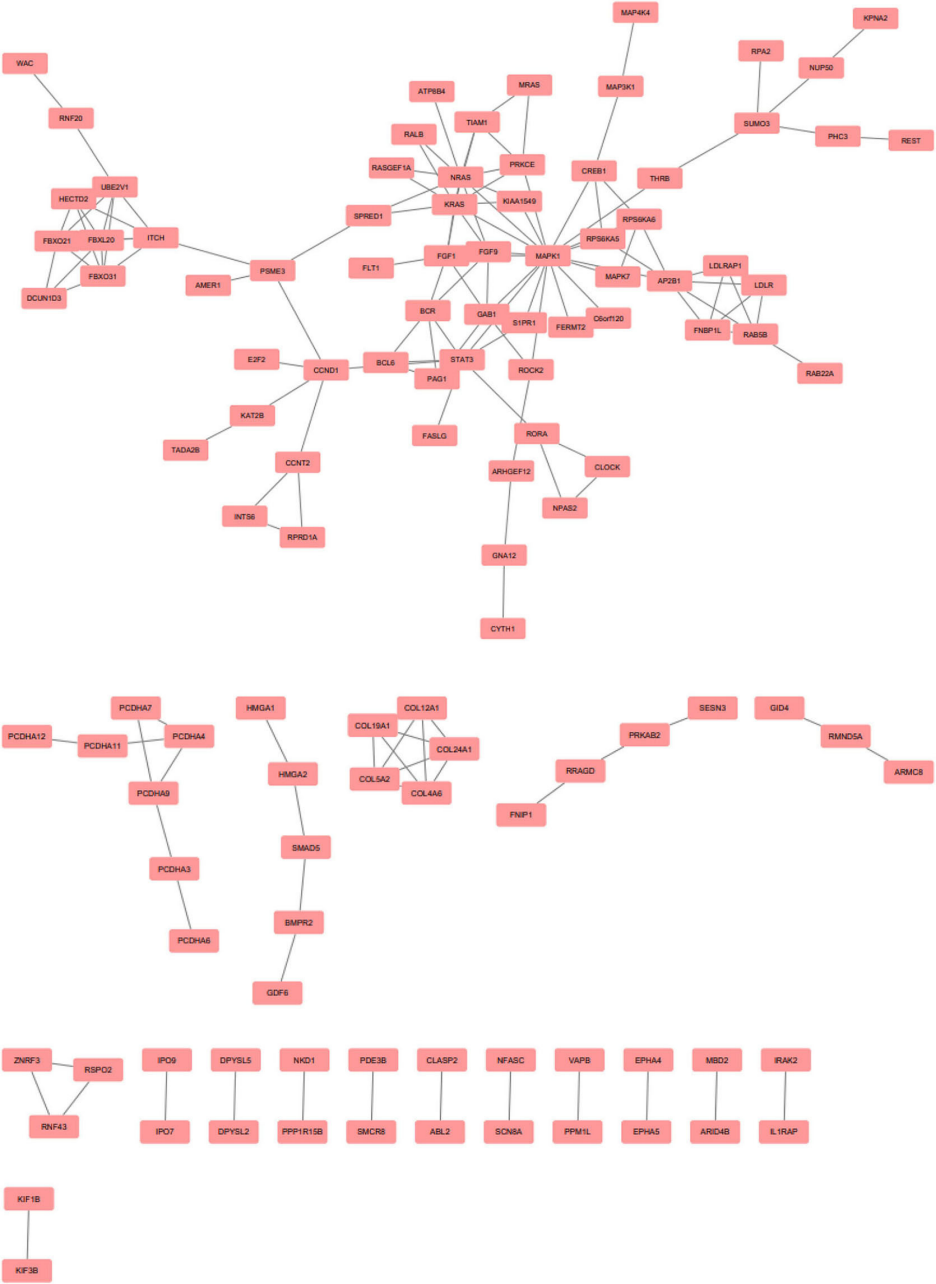
Protein-protein interaction network analysis of the target genes of downregulated microRNAs. Gene interactions were constructed using the STRING online database and Cytoscape. Network nodes represent genes, and the edges represent protein-protein associations.

EnrichR is a free web-based gene signature search tool. It was used to evaluate the 247 target genes of upregulated miRNAs, among which 573 terms were identified. Among the 822 target genes of downregulated miRNAs, 1,017 terms were found. EnrichR provides a visualization summary of the pathways based on a collective gene function list. The free pathway database tool Reactome is available for online use, and provides biological interpretation and visualization models for network analysis. STRING analysis was performed on the target genes of upregulated miRNAs (Figure 3) and downregulated miRNAs (Figure 4). The results were visualized using Cytoscape.

## Discussion

### Differentially Expressed miRNAs

MiRNAs can act as oncogenes or tumor suppressor genes in CML, contributing to the pathogenesis, disease progression, and therapeutic responses (1, 52). Following the advent of TKIs as specific target drugs, hematopoietic stem cell transplantation began to play an important role in treating CML patients that are in the disease phase or are resistant to TKIs. This study investigated the miRNA profile of a group of 14 CML patients treated with allo-HSCT, who were in complete cytogenetic remission at the time of sample collection. Among the evaluated group, five patients underwent transplantation due to disease progression (patients 1, 3, 7, 10, and 14) and four others underwent transplantation due to a failed therapeutic response (patients 2, 5, 8, and 9). These comprised a total of 64.3% of the evaluated patients.

### miR-10a

MiR-10a has been extensively studied due to its potential as a CML marker. Flamant et al. suggested the relevance of miR-10a as a drug response biomarker. By means of microarray analysis, a significant increase in miR-10a was observed in patient samples 2 weeks post-imatinib treatment (34). miR-10a downregulation was also detected in the transplanted patient group in the present study.

### miR-17/92 Cluster

The miR-17/92 cluster is another potential biomarker for CML progression, and may be detected from the chronic phase to the blastic phase. This cluster is comprised of miR-17, miR18a, miR-19a, miR19b-1, miR20a, and miR92a-1 (32). In our study, a downregulation in the expression of miR-18a and miR-19a was observed, both of which are present in this cluster.

### miR-328 and miR150

miR-328 and miR-150 are also related to disease progression (32, 48, 54, 64). Eiring et al. (48) have previously demonstrated the loss of miR-328 in blastic crisis CD34^+^ cells, which did not otherwise occur in chronic phase CD34^+^ myeloid cells. Poláková et al. (52) detected a negative correlation between the levels of *BCR-ABL* transcript and miR-150, further substantiating previous findings by Agirre et al. (64) and Fallah et al. (54) suggested that the downregulation of miR-150 is a potential diagnostic marker of CML. RT-PCR was performed for 50 samples from patients newly diagnosed with CML, revealing a downregulation in miR-150. In our study, both miRNAs were found to be upregulated, suggesting that miR-328 and miR-150 are useful as molecular biomarkers of the treatment response.

### miR-486

The results of previous studies have also suggested miR-486 as an effective treatment response marker. Wang et al. compared miRNA expression in normal human CD34^+^ cells with that in CML CD34^+^ cells, using human leukemia cell lines and CD34^+^ cells isolated from chronic phase CML patients who had not been treated with imatinib. The results showed that miR-486 was overexpressed in CML cells, especially in megakaryocytes and erythroid progenitor cells (36). In our study, miR-486 was downregulated, further suggesting the potential of this miRNA to act as a biomarker of the treatment response.

### miR-181c and miR-30

According to studies carried out in the K562 cell line, miR-181c, and miR-30 downregulation is related to imatinib resistance (30, 31). Yu et al. demonstrated that the downregulation of miR-30 in this CML cell line was related to the autophagy process and imatinib resistance mechanisms (30, 31). These results were reflected in the current study, with our findings demonstrating that miR-181c and miR-30 were downregulated.

### Let-7g and miR-16

Let-7a and miR-16 also have important functions in myeloid leukemogenesis, such as regulating the cell cycle and apoptosis. Let-7a regulates oncogenes such as RAS and HMGA, while Mir-16 targets MCL1, BCL2, WNT3A, and CCNDI (35). Let-7a is also a member of the let-7 family, and it has been found that it may suppress CML via *CRKL* (20). Zuo et al. (35) examined the plasma levels of miR-16 and let-7a in a group of 50 patients with myelodysplastic syndrome (MDS). After comparing these results with those from a group of 76 healthy donors, it was found that both miRNAs were significantly lower in MDS patients than in healthy controls. In the present study, we found that let-7g and miR-16 were downregulated.

### Pathway Enrichment Analysis and PPI Network Construction

In the analysis conducted using STRING and EnrichR, it was possible to determine which pathways were enriched by observing the target genes of unregulated miRNAs. Through our analysis, we found that MAPK, NRAS, KRAS, and ROCK had important functions related to CML regulation pathways.

The mitogen-activated protein kinase (MAPK) pathway is an important signaling cascade in several types of cancer, including CML (65). This pathway controls fundamental cellular processes such as cell proliferation, migration, growth, differentiation, and death (66). The MAPK pathway therefore plays a fundamental role in cell growth and survival, and irregularities in this pathway can lead to cells developing cancerous properties. This may include exacerbated cell proliferation, metastasis, and evasion of apoptosis (67).

RAS proteins are involved in signal transduction, and are mutated in different types of human cancer. The RAS family comprises three genes associated with carcinogenesis: HRAS, KRAS, and NRAS (68). These three genes encode a protein located on the inner cell membrane, which has GTPase activity. This protein participates in extracellular signal transduction into the cell, and the signal is transmitted by a cascade of kinases. As a result, MAPK is activated, which then activates transcription factors. Mutations in the RAS genes in human cancer inhibit GTP hydrolysis, and mutated RAS proteins remain in their GTP-linked active form. This, leads to disordered cell proliferation (69). KRAS and NRAS mutations are frequently found in myeloid disorders, including CML (70).

ROCK proteins perform different functions in cells. They are involved in organization of the actin cytoskeleton, human tumor pathogenesis, and in the signaling pathways that lead to cell proliferation. The RAS and ROCK pathways are interconnected, with RAS activating PI3K, which then activates the ROCK pathway. This interaction network can lead to continuous cell proliferation and survival (68).

The MAPK, RAS, and ROCK genes had increased expression in the patient group of our study. As these genes are involved in cell proliferation during leukemogenesis, it can be concluded that these genes play an important role in post-transplant evolution. Nine of the 14 patients (64.3%) had leukemia relapse, and the other five (35.7%) did not achieve a deep molecular response (BCR-ABL transcript level ≤ 0.01%).

This study explored miRNAs in CML patients and their target genes, and analyzed the pathways involved. Our study was based on original and clinical samples. However, as there were scarce samples available and these were insufficient to validate the 46 altered miRNAS, the study was limited by this factor. The bioinformatics analyses predicted interactions that require further biological validation before their therapeutic application.

Despite the small sample size, these findings showed that aggressive therapy such as transplantation did not alter the disease course in these group of CML patients. Furthermore, the demonstrated pattern of gene expression is suggestive of disease progression.

## Data Availability Statement

All datasets generated for this study are included in the article/Supplementary Material.

## Ethics Statement

This study was reviewed and approved by Research Ethics Committee of Dr. Amaral Carvalho Hospital–Jahu-SP- Brazil (CEPHAC—2.917.389). The patients/participants provided their written informed consent to participate in this study.

## Author Contributions

PH, NH, and CN designed and coordinated the study and prepared the manuscript. JM, LM, and SC performed bioinformatics analysis and prepared the manuscript. JD and JC collected and analyzed the data. RC offered laboratory support and helped with discussions about the study. All authors critically reviewed and approved the final version of the manuscript.

## Conflict of Interest

The authors declare that the research was conducted in the absence of any commercial or financial relationships that could be construed as a potential conflict of interest. The handling editor declared a shared affiliation, though no other research collaboration, with the authors.
