# Supplementary material for: Comparison of microRNA Expression Profile in Chronic Myeloid Leukemia Patients Newly Diagnosed and Treated by Allogeneic Hematopoietic Stem Cell Transplantation
Source: Front Oncol. 2020 Sep 4;10:1544. doi: 10.3389/fonc.2020.01544 (PMC7500210; doi:10.3389/fonc.2020.01544)
Supplement: Supplementary file 1 [file Table_1.docx]

**TABLE 1| Patient clinical data**

**Untreated Group**

| **Patients** | **Gender Age** | **BCR-ABL (%)** | **Breakpoint** |  |
| --- | --- | --- | --- | --- |
| 1 M 61 92 b2a2  2 M 52 100 b2a2  3 M 35 100 b2a2  4 F 54 100 b2a2  5 M 46 73 b3a2  6 M 72 100 b2a2  7 F 80 100 b3a2  8 M 67 100 b3a2  9 M 59 40 b2a2  10 M 60 82 b2+b3  11 F 52 100 b3a2  12 M 37 100 b3a2  13 F 53 100 b3a2  14 M 72 100 b3a2 | | | | |

**Hematopoietic Stem Cell Transplantation Group**

| **Patients** | **Gender** | **Age BCR-ABL (%)** | **DAT Breakpoint Source BCR-ABL kinase domain mutation** |
| --- | --- | --- | --- |
| 1 M 37 1,60 224 b2a2 BM absence  2 F 44 1,20 231 b3a2 BM absence  3 F 03 0,20 208 b3a2 BM -  4 F 63 0,20 1569 b3a2 BM absence  5 M 37 0,50 110 b2a2 BM -  6 M 43 0,30 2450 b3a2 BM -  7 M 48 0,75 80 b3a2 PBSC absence  8 M 35 0,03 2352 b3a2 BM absence  9 M 42 0,01 3561 b2a2 PBSC -  10 F 42 0,01 3708 b3a2 PBSC -  11 F 56 0,03 3932 b2a2 BM absence  12 M 32 0,08 3242 b2a2 PBSC -  13 M 21 0,30 1949 b3a2 BM absence  14 M 58 0,02 41 b2a2 BM - | | | |

DAT: days after transplantation; BM: bone marrow; PBSC: peripheral blood stem cell, (-) BCR-ABL mutation test was not performed.
