# Supplementary material for: Comparison of microRNA Expression Profile in Chronic Myeloid Leukemia Patients Newly Diagnosed and Treated by Allogeneic Hematopoietic Stem Cell Transplantation
Source: Front Oncol. 2020 Sep 4;10:1544. doi: 10.3389/fonc.2020.01544 (PMC7500210; doi:10.3389/fonc.2020.01544)
Supplement: Supplementary file 2 [file Table_2.docx]

**TABLE 2**| Dysregulated miRNAs in chronic myeloid leukemia

| **miR Name** | **miRBase ID** | **FC** | **p-value** |  |
| --- | --- | --- | --- | --- |
| hsa-miR-1260a | [MIMAT0005911](http://mirbase.org/cgi-bin/mature.pl?mature_acc=MIMAT0005911) | **0,013** | 0.000 |  |
| hsa-miR-27a-3p | [MIMAT0000084](http://mirbase.org/cgi-bin/mature.pl?mature_acc=MIMAT0000084) | **0,082** | 0.000 |  |
| hsa-miR-140-3p | [MIMAT0004597](http://mirbase.org/cgi-bin/mature.pl?mature_acc=MIMAT0004597) | **0,083** | 0.000 |  |
| mmu-miR-374b-5p | [MIMAT0003727](http://mirbase.org/cgi-bin/mature.pl?mature_acc=MIMAT0003727) | **0,105** | 0.000 |  |
| hsa-miR-143-3p | [MIMAT0000435](http://mirbase.org/cgi-bin/mature.pl?mature_acc=MIMAT0000435) | **0,150** | 0.000 |  |
| hsa-miR-181c-5p | [MIMAT0000258](http://mirbase.org/cgi-bin/mature.pl?mature_acc=MIMAT0000258) | **0,159** | 0.000 |  |
| hsa-miR-26b-5p | [MIMAT0000083](http://mirbase.org/cgi-bin/mature.pl?mature_acc=MIMAT0000083) | **0,160** | 0.000 |  |
| hsa-miR-212-3p | [MIMAT0000269](http://mirbase.org/cgi-bin/mature.pl?mature_acc=MIMAT0000269) | **0,164** | 0.000 |  |
| hsa-miR-29c-3p | [MIMAT0000681](http://mirbase.org/cgi-bin/mature.pl?mature_acc=MIMAT0000681) | **0,172** | 0.000 |  |
| hsa-miR-26a-1-3p | [MIMAT0004499](http://mirbase.org/cgi-bin/mature.pl?mature_acc=MIMAT0004499) | **0,180** | 0.000 |  |
| hsa-miR-181a-5p | [MIMAT0000256](http://mirbase.org/cgi-bin/mature.pl?mature_acc=MIMAT0000256) | **0,189** | 0.000 |  |
| hsa-miR-19a-3p | [MIMAT0000073](http://mirbase.org/cgi-bin/mature.pl?mature_acc=MIMAT0000073) | **0,224** | 0.008 |  |
| hsa-miR-363-3p | [MIMAT0000707](http://mirbase.org/cgi-bin/mature.pl?mature_acc=MIMAT0000707) | **0,243** | 0.001 |  |
| hsa-miR-30d-5p | [MIMAT0000245](http://mirbase.org/cgi-bin/mature.pl?mature_acc=MIMAT0000245) | **0,252** | 0.000 |  |
| hsa-miR-10a-5p | [MIMAT0000253](http://mirbase.org/cgi-bin/mature.pl?mature_acc=MIMAT0000253) | **0,259** | 0.014 |  |
| hsa-miR-29a-3p | [MIMAT0000086](http://mirbase.org/cgi-bin/mature.pl?mature_acc=MIMAT0000086) | **0,291** | 0.000 |  |
| hsa-miR-16-5p | [MIMAT0000069](http://mirbase.org/cgi-bin/mature.pl?mature_acc=MIMAT0000069) | **0,291** | 0.002 |  |
| hsa-miR-486-5p | [MIMAT0002177](http://mirbase.org/cgi-bin/mature.pl?mature_acc=MIMAT0002177) | **0,292** | 0.029 |  |
| hsa-miR-345-5p | [MIMAT0000772](http://mirbase.org/cgi-bin/mature.pl?mature_acc=MIMAT0000772) | **0,310** | 0.000 |  |
| hsa-miR-26a-5p | [MIMAT0000082](http://mirbase.org/cgi-bin/mature.pl?mature_acc=MIMAT0000082) | **0,318** | 0.000 |  |
| hsa-miR-18a-3p | [MIMAT0002891](http://mirbase.org/cgi-bin/mature.pl?mature_acc=MIMAT0002891) | **0,329** | 0.000 |  |
| hsa-miR-27b-3p | [MIMAT0000419](http://mirbase.org/cgi-bin/mature.pl?mature_acc=MIMAT0000419) | **0,331** | 0.003 |  |
| hsa-miR-374a-5p | [MIMAT0000727](http://mirbase.org/cgi-bin/mature.pl?mature_acc=MIMAT0000727) | **0,343** | 0.009 |  |
| hsa-miR-362-5p | [MIMAT0000705](http://mirbase.org/cgi-bin/mature.pl?mature_acc=MIMAT0000705) | **0,350** | 0.000 |  |
| hsa-let-7g-5p | [MIMAT0000414](http://mirbase.org/cgi-bin/mature.pl?mature_acc=MIMAT0000414) | **0,402** | 0.000 |  |
| hsa-miR-324-3p | [MIMAT0000762](http://mirbase.org/cgi-bin/mature.pl?mature_acc=MIMAT0000762) | **0,417** | 0.000 |  |
| hsa-miR-550a-5p | [MIMAT0004800](http://mirbase.org/cgi-bin/mature.pl?mature_acc=MIMAT0004800) | **0,417** | 0.003 |  |
| hsa-miR-125a-3p | [MIMAT0004602](http://mirbase.org/cgi-bin/mature.pl?mature_acc=MIMAT0004602) | **0,444** | 0.046 |  |
| hsa-miR-106b-5p | [MIMAT0000680](http://mirbase.org/cgi-bin/mature.pl?mature_acc=MIMAT0000680) | **0,477** | 0.001 |  |
| hsa-miR-191-5p | [MIMAT0000440](http://mirbase.org/cgi-bin/mature.pl?mature_acc=MIMAT0000440) | **0,496** | 0.000 |  |
| hsa-miR-15b-3p | [MIMAT0004586](http://mirbase.org/cgi-bin/mature.pl?mature_acc=MIMAT0004586) | **2,005** | 0.039 |  |
| hsa-miR-328-3p | MIMAT0000752 | **2,179** | 0.013 |  |
| hsa-miR-222-3p | [MIMAT0000279](http://mirbase.org/cgi-bin/mature.pl?mature_acc=MIMAT0000279) | **2,233** | 0.000 |  |
| hsa-miR-139-5p | [MIMAT0000250](http://mirbase.org/cgi-bin/mature.pl?mature_acc=MIMAT0000250) | **2,338** | 0.039 |  |
| hsa-miR-92a-3p | [MIMAT0000092](http://mirbase.org/cgi-bin/mature.pl?mature_acc=MIMAT0000092) | **2,492** | 0.002 |  |
| hsa-miR-628-3p | [MIMAT0003297](http://mirbase.org/cgi-bin/mature.pl?mature_acc=MIMAT0003297) | **2,589** | 0.010 |  |
| hsa-miR-150-5p | [MIMAT0000451](http://mirbase.org/cgi-bin/mature.pl?mature_acc=MIMAT0000451) | **2,748** | 0.034 |  |
| hsa-miR-574-3p | [MIMAT0003239](http://mirbase.org/cgi-bin/mature.pl?mature_acc=MIMAT0003239) | **2,764** | 0.001 |  |
| hsa-miR-484 | [MIMAT0002174](http://mirbase.org/cgi-bin/mature.pl?mature_acc=MIMAT0002174) | **2,820** | 0.000 |  |
| hsa-miR-127-3p | [MIMAT0000446](http://mirbase.org/cgi-bin/mature.pl?mature_acc=MIMAT0000446) | **3,969** | 0.007 |  |
| hsa-miR-146a-5p | [MIMAT0000449](http://mirbase.org/cgi-bin/mature.pl?mature_acc=MIMAT0000449) | **3,973** | 0.000 |  |
| hsa-miR-193a-5p | [MIMAT0004614](http://mirbase.org/cgi-bin/mature.pl?mature_acc=MIMAT0004614) | **4,513** | 0.000 |  |
| hsa-miR-342-3p | [MIMAT0000753](http://mirbase.org/cgi-bin/mature.pl?mature_acc=MIMAT0000753) | **5,070** | 0.000 |  |
| hsa-miR-7-1-3p | [MIMAT0004553](http://mirbase.org/cgi-bin/mature.pl?mature_acc=MIMAT0004553) | **5,650** | 0.000 |  |
| mmu-miR-134-5p | [MIMAT0000146](http://mirbase.org/cgi-bin/mature.pl?mature_acc=MIMAT0000146) | **6,473** | 0.002 |  |
| hsa-miR-409-3p | [MIMAT0001639](http://mirbase.org/cgi-bin/mature.pl?mature_acc=MIMAT0001639) | **10,905** | 0.004 |  |

______________________________________________________________

FC, fold-change; miR, microRNA.
