# Supplementary material for: Comparison of microRNA Expression Profile in Chronic Myeloid Leukemia Patients Newly Diagnosed and Treated by Allogeneic Hematopoietic Stem Cell Transplantation
Source: Front Oncol. 2020 Sep 4;10:1544. doi: 10.3389/fonc.2020.01544 (PMC7500210; doi:10.3389/fonc.2020.01544)
Supplement: Supplementary file 3 [file Table_3.docx]

**TABLE 3**| Functions of dysregulated miRNAs in chronic myeloid leukemia

| **miR Name Dysregulation in cancer References** |
| --- |
| hsa-miR-1260a Down-regulated in follicular B-cell lymphoma [31] |
| hsa-miR-27a-3p Involved in tumor growth: cell proliferation and cell invasion [34] |
| hsa-miR-140-3p Chemoresistance in osteosarcoma and colon cancer [35] |
| mmu-miR-374b-5p Inhibits cell migration, proliferation and invasion in cervical  cancer [36] |
| hsa-miR-143-3p Low expression level contributes to tumor development,  differentiation, proliferation, invasion and metastasis [37] |
| hsa-miR-181c-5p Inhibits chemoresistance in chronic myelocytic leucemia [38] |
| hsa-miR-26b-5p Down-regulated in breast cancer [39] |
| hsa-miR-212-3p Inhibits cell proliferation and promotes apoptosis [40] |
| hsa-miR-29c-3p Deregulated in hematological malignances [41] |
| hsa-miR-26a-1-3p Tumor supressor [42] |
| hsa-miR-181a-5p Downregulation in resistance to imatinib [27,28] |
| hsa-miR-19a-3p Potential biomarker for CML [22] |
| hsa-miR-363-3p Tumor suppressor in gastric cancer [43] |
| hsa-miR-30d-5p Downregulation in resistance to imatinib [27,28] |
| hsa-miR-10a-5p Biomarker of drug response in CML [20] |
| hsa-miR-29a-3p Deregulated in hematological malignances [41] |
| hsa-miR-16-5p Regulation of cell cycle and apoptosis in myeloid leukemogenesis [29] |
| hsa-miR-486-5p Expression increased in erythroid differentiation in CML [26] |
| hsa-miR-345-5p Tumor supressor in pancreatic cancer [44] |
| hsa-miR-26a-5p Tumor supressor [41] |
| hsa-miR-18a-3p Potential biomarker for CML [23] |
| hsa-miR-27b-3p Oncogene; expression increased in lymphoma [45] |
| hsa-miR-374a-5p Promotes proliferation and migration of transformed  mesenchymal stem cells [46] |
| hsa-miR-362-5p Induces apoptosis resistance and cell proliferation in gastric cancer [47] |
| hsa-let-7g-5p Downregulated in Burkitt´s lymphoma [48] |
| hsa-miR-324-3p Overexpression promotes cell growth and decreases apoptosis [49] |
| hsa-miR-550a-5p Tumor supressor [50] |
| hsa-miR-125a-3p Induces apoptosis in pancreatic cancer [51] |
| hsa-miR-106b-5p Inhibits metastasis and invasion colorectal cancer cells [52] |
| hsa-miR-191-5p Disregulated in human gliobastoma tissues [53] |
| hsa-miR-15b-3p High expression in poor prognosis for hepatocellular carcinoma [54] |
| hsa-miR-328-3p CML progression [24] |
| hsa-miR-222-3p Cancer development as oncomiR or as oncosupressor [55] |
| hsa-miR-139-5p Antimetastic and anti-oncogenic activity [56] |
| hsa-miR-92a-3p Higher levels in acute myeloid leukemia and acute lymphoblastic  leukemia [57] |
| hsa-miR-628-3p Inhibits proliferation of acute myeloid leukemia cells [19,58] |
| hsa-miR-150-5p CML progression; CML biomarker [25] |
| hsa-miR-574-3p Tumor suppressor in ovarian cancer [59] |
| hsa-miR-484 Highly expressed in breast cancer patients [60] |
| hsa-miR-127-3p Tumor suppressors in gastric cancer [61] |
| hsa-miR-146a-5p Development and maintenance of neoplastic processes [62] |
| hsa-miR-193a-5p Low expression in lung cancer [63] |
| hsa-miR-342-3p Suppresses acute myeloid leukemia cell proliferation [64] |
| hsa-miR-7-1-3p Up-regulated in metastatic prostate cancer [65] |
| mmu-miR-134-5p Cancer cell proliferation [65] |
| hsa-miR-409-3p Tumor supressor in endometrial carcinoma cells [66] |

____________________________________________________________________

Fc, fold change; miR, microRNA.
