# Supplementary figures and images for: Comparison of microRNA Expression Profile in Chronic Myeloid Leukemia Patients Newly Diagnosed and Treated by Allogeneic Hematopoietic Stem Cell Transplantation
Source: Front Oncol. 2020 Sep 4;10:1544. doi: 10.3389/fonc.2020.01544 (PMC7500210; doi:10.3389/fonc.2020.01544)

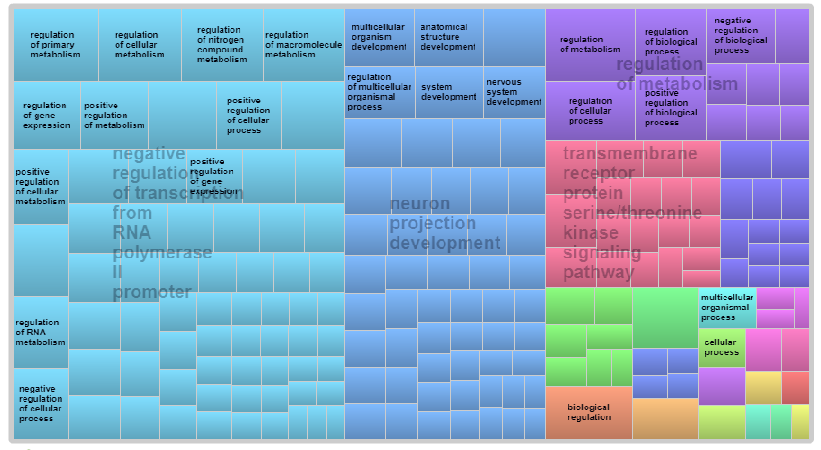

Supplement: Supplementary file 5 [file Table_5.DOCX]
